# Associations Between Acculturation, Depressive Symptoms, and Life Satisfaction Among Migrants of Turkish Origin in Germany: Gender- and Generation-Related Aspects

**DOI:** 10.3389/fpsyt.2020.00715

**Published:** 2020-07-30

**Authors:** Eva Morawa, Tilman Brand, Nico Dragano, Karl-Heinz Jöckel, Susanne Moebus, Yesim Erim

**Affiliations:** ^1^ Department of Psychosomatic Medicine and Psychotherapy, University Hospital Erlangen, Friedrich-Alexander University Erlangen-Nürnberg (FAU), Erlangen, Germany; ^2^ Department for Prevention and Evaluation, Leibniz Institute for Prevention Research and Epidemiology – BIPS, Bremen, Germany; ^3^ Institute for Medical Sociology, University Hospital Düsseldorf, Düsseldorf, Germany; ^4^ Institute for Medical Informatics, Biometry and Epidemiology (IMIBE), University Hospital Essen, Essen, Germany; ^5^ Institute for Urban Public Health, University Hospital Essen, Essen, Germany

**Keywords:** depression, life satisfaction, mental health, migrants, Turkish

## Abstract

**Objective:**

The aim of the present study was to investigate the degree of depressive symptoms and life satisfaction (LS) as well as the association between acculturation and depressive symptoms among a large sample of persons with Turkish migration background in Germany, taking into account gender- and migration-related differences.

**Methods:**

This study was part of a pretest for a large national epidemiological cohort study in Germany. Acculturation was measured using the Frankfurt Acculturation Scale (FRACC). Based on the median split of the two subscales, four acculturative styles according to Berry (integration, assimilation, separation and marginalization) were determined. Depressive symptoms were assessed with the depression module (PHQ-9) from the Patient Health Questionnaire. LS was assessed with a single item on a scale from 1 = bad to 5 = excellent. Differences in levels of depressive symptoms and LS in relation to gender and generation of migration were tested with analysis of covariance, controlling for age. Gender-stratified multiple linear regression analyses were also conducted for depressive symptoms as criterion variable.

**Results:**

328 Turkish migrants participated (61.3% women). The cut-off-value of ≥10 for the PHQ-9 was achieved by 33.2% of the women and 26.4% of the men (p=0.209, φ=0.071). In female migrants, the age-adjusted mean score for depressive symptoms was 7.81 (SD=6.42), in males 6.70 (SD=6.41) (p=0.137, η^2^=0.007). After controlling for unemployment status, women showed a trend for being more frequently depressed than men (p=0.055, φ=0.117) and also demonstrated a trend for higher levels of depressive symptoms (p=0.072, η^2^=0.012). No significant gender-specific difference was found concerning age-adjusted mean score for LS (p=0.547, η^2^=0.001), also when controlled for unemployment status (p=0.322, η^2^=0.004). In both sexes, the second generation demonstrated a significantly higher age-adjusted mean score for LS of small/medium effect size than the first generation. In women, separation as acculturation style (linear regression coefficient (B=4.42, 95% CI=1.68, 7.17, p=0.002; reference: integration), having no partnership (B=2.56, 95% CI=0.26, 4.86, p=0.03) and lower education (B=-2.28, 95% CI=-4.54, -0.02, p=0.048) were associated with higher severity of depressive symptoms; in men, separation as acculturation style (B=4.01, 95% CI=0.70, 7.31, p=0.018; reference: integration) and employment status (B=-3.32, 95% CI=-5.71, -0.92, p=0.007) were related to depression levels.

**Conclusions:**

Separation as acculturation style is associated with higher levels of depressive symptoms (for both genders). Gender-sensitive health promotion programs should target separated migrants to improve their integration into the German society.

## Introduction

In the face of expanding numbers of migrants, knowledge regarding the influence of culture-specific and migration-related factors on mental health presents a substantial issue for research in the public health sector. In Germany, every fourth person (25.5%) of the population has a migration background, either having personally moved there or having at least one parent who immigrated (1). With 2.8 million, individuals of Turkish origin constitute the largest ethnic group in this country (1).

Population-based health surveys in Germany (2), the Netherlands (3), and Belgium (4) as well as clinical studies have consistently demonstrated higher prevalence rates of depression and increased levels of depressive symptoms in Turkish migrants in comparison with the majority populations and other migrant collectives (5–7). Women of Turkish origin frequently demonstrate higher levels of depressive complaints than men (8, 9) and also higher prevalence rates (3). Several socio-demographic risk factors for depression in Turkish migrants have also been identified: older age (9, 10), a low socio-economic status (9, 11), unemployment (12), no current partnership (10), belonging to the first migration generation (13); however, other studies found no significant differences between generations (4, 10).

The migration into a host country as well as the post-migration acculturation process is associated with various stressors. Acculturation is a complex, multifaceted, and long-term process of psychological and social changes resulting from continuous interaction between individuals from different cultures (14). The most renowned theory on acculturation is the one presented by Berry (14), which proposes four acculturation styles: integration, assimilation, separation, and marginalization. In the majority of studies on the association between acculturation and depression conducted among Turkish migrants living in Germany or other European countries as well as among other migrant populations, the acculturation style of integration or assimilation (13, 15–17) was often associated with a better mental health status, whereas poor outcomes were consistently predicted by marginalization (16, 18).

While depression represents a negative indicator for affective well-being, life satisfaction (LS) stands for the cognitive well-being (19). LS is defined as a person´s global evaluation of contentment with life (20). It involves all aspects that determine the individual quality of life, such as health-and job-related, social, financial or other factors. Previous international research on LS in migrants reveals inconsistent findings. Some population-based studies have identified lower LS among migrants in comparison with the majority population, in particular for the second generation (21) or for the first generation migrants (22), however, also greater levels of LS in migrants (23) or similar LS have been reported (24). A population-based survey in Germany showed lower LS in the migrants as compared with native-born Germans, however the effect size was minimal; migrants from Turkey reported the lowest LS (25). Also in other Western countries a lower extent of LS has been identified in Turkish migrants in relation to the majority population – so for example in the Netherlands (26). In a study examining migrant mothers of Turkish origin in Germany, a higher socioeconomic status was related to higher LS (11). Among women of Turkish origin living in Great Britain, the integration style and higher religious identity was found to be associated with higher LS (27).

Due to the lack of gender-stratified analyses with persons of Turkish origin in Germany in population-based surveys or large samples as well as inconclusive findings, the central objective of the present study was to examine the association between acculturation and depressive symptoms among women and men of Turkish origin in Germany. Acculturation was analyzed by applying a categorization of four acculturation styles (integration, assimilation, separation, and marginalization). The aims of the study were:

1. to examine the differences regarding the frequency and severity of depressive symptoms and the degree of LS between women and men of Turkish origin and the first and second migration generation (gender-stratified);
2. to explore the association between acculturative styles and the severity of depressive symptoms when adjusted for socio-demographic and migration-related variables separately in women and men of Turkish origin.

Based on prior research, we hypothesized higher levels of depressive symptoms and lower LS to be prevalent in women and in the first generation. Furthermore, we postulated separation (according Berry) and marginalization to be associated with higher levels of depressive symptoms in reference to integration and no significant difference between assimilation and integration.

## Materials and Methods

### Sample Description and Procedure

Participants were recruited in the city of Essen (North Rhine Westphalia) between December 2011 and August 2012 using two recruitment methods: a community-orientated and a register-based strategy [for details see (28, 29)]. Within the community-orientated approach, the representatives of the Turkish community (e.g., academic institutions, medical practices, religious institutions) supported the recruitment of persons of Turkish origin as key persons. Furthermore, the social networks of Turkish migrants were contacted for recruitment such as mosques, Turkish speaking general practitioners and doctors of other medical professions, Turkish parents´ and teachers´ associations, as well as other associations, Workers´ Welfare Organization, neighbors or relatives of the participants. In addition, the investigation was widely promoted in the Turkish community of Essen by word-of-mouth recommendation. Persons interested in participation in the study contacted the study center by phone. 319 persons of Turkish origin participated in the study as a result of the community-orientated recruitment. Within the register-based approach, a random sample of 1498 potential participants with Turkish citizenship from the Essen population registry was extracted and potential participants were sent a written invitation for the study in both Turkish and German. In the case of no response within approximately two weeks a second invitation letter, after further two weeks a third letter were sent. 286 participants were recruited by the register-based method. The response rate for the register-based approach was 19.4%.

Inclusion criteria for the study were: age between 20 and 69 years [due to the design of the German National Cohort Study (30)], agreement to participate in the study, the status of a person of (Turkish) migration background according to the definition applied in epidemiological research in Germany (1) (having either immigrated themselves = first generation migrants or having at least one parent who immigrated or were born as non-German in Germany = second generation migrants), and principal residence in Essen.

The investigation consisted of different medical examinations and a set of self-report questionnaire including a self-administered questionnaire on mental health issues. Questionnaires were available in Turkish and German. Participants were asked to complete the mental health module at home and send it back to the examination office. For further details see Reiss et al. (28). If there was no response to the questionnaires of the mental health module within the designated time period, the participants were contacted by phone.

The data have already been used for other publications focusing on somatic symptoms/somatization among Turkish migrants (29) and the association between acculturation and health-related quality of life (18). There is a topical overlap with our previous published work (13) concerning the relationship between acculturation and depressive symptoms, however in our prior work we have investigated clinical samples, whereas the present study has examined a non-clinical sample. The additional knowledge provided by this study results from the gender-stratified analyses allowing the investigation of gender-specific differences. The advantage of the present sample is also based on its composition (healthy participants). Therefore, a sample selection bias may be reduced.

### Ethics Statement

The present study was approved by the Ethics Committee of the Medical Faculty of the University of Duisburg-Essen (reference number: 11-4861-BO). Written informed consent was obtained from all participants.

### Measures

#### Socio-Demographic and Migration-Specific Variables

The following socio-demographic characteristics of the participants were assessed: gender, age, partnership, marital status, education level, employment status, and monthly household income.

The following migration-specific variables were assessed: if born in Germany or length of residence in Germany and age at time of migration into Germany, citizenship, and language proficiency.

#### Patient Health Questionnaire: Depression Module (PHQ-9)

Depressive symptoms were assessed with the depression module of the Patient Health Questionnaire (PHQ-9) (31). It consists of 9 items and is based on DSM-IV criteria. The sum scores range from 0 to 27 representing mild, moderate and severe levels of depressive symptomatology with cut-off-points of ≥5, ≥10, and ≥15. The psychometric characteristics of the PHQ-9 are well documented (32). The internal consistency (Cronbach´s Alpha) for the German version is 0.86–0.89 (31) and for the Turkish version 0.86 (33). In the present sample the validated German version obtained a Cronbach´s Alpha of 0.91, the Turkish version a Cronbach´s Alpha of 0.89.

#### Frankfurt Acculturation Scale (FRACC)

The acculturation strategies were measured with the Frankfurt Acculturation Scale (FRACC) (34), a self-report questionnaire comprising 20 items rated on a seven-point Likert scale (0 = absolutely not to 6 = absolutely). The questionnaire consists of two indices (each with 10 items and values between 0 and 60 for each index) assessing the degree of orientation towards culture of origin (CO), and the degree of orientation towards the host culture (HC). Higher scores indicate a higher orientation towards the CO or HC, respectively. In the present study the Cronbach’s Alpha for the Turkish version was 0.75 for CO and 0.53 for HC and for the German version 0.86 for CO and 0.79 for HC.

The medians of both indices of the acculturation scale were used as cut-off points to divide the sample into a group of subjects with a low CO vs. a group with a high CO, as well as a group of subjects with a low HC vs. a group with a high HC. We categorized the participants into four groups (= acculturation styles) based on the schema of Berry (35): integration (CO-high and HC-high), assimilation (HC-high and CO-low), separation (CO-high and HC-low), and marginalization (CO-low and HC-low).

#### Life Satisfaction

General life satisfaction in Germany was assessed with a single item on a scale from 1 = bad to 5 = excellent.

### Statistical Analysis

Data analyses were conducted with SPSS V. 21. Missing values in the questionnaires were replaced by the expectation-maximization algorithm (max. 30% missing data per questionnaire was accepted, otherwise the case was excluded from the analysis). Descriptive statistics (means, standard deviations, ranges, and frequencies) were computed to profile the socio-demographic and migration-specific sample characteristics.

To explore the differences regarding age and gender between the non-respondents from the population registry and the participants recruited with the register-based strategy, a t-test for independent samples and a binomial test were performed, respectively. Gender differences in the frequency of being depressed (cut-off-value PHQ-9≥10) and the association between the acculturative styles and the language proficiency (dichotomized) were tested with the χ^2^-test.

An analysis of covariance (ANCOVA) was calculated to examine the differences in degree of depressive symptoms in relation to relevant socio-demographic (gender) and migration-related characteristics (generation of migration), controlling for age. The effect sizes (η^2^ and φ) were also reported (η^2^ ≥ 0.01 = small, η^2^ ≥ 0.06 = medium und η^2^ ≥ 0.14 = large effect size; φ ≥ 0.1 = small, φ ≥ 0.3 = medium und φ ≥ 0.5 = large effect size) (36). Gender-stratified multiple linear regression analyses with enter method were performed to investigate the influence of the acculturation styles as well as socio-demographic and migration-related characteristics on the severity of depressive symptoms in women and men. In case of missing values, the pairwise exclusion was used. Multicollinearity was checked by calculating the variance inflation factors. A level of significance of p<.05 (two-tailed) was predetermined in all analyses.

## Results

A total of 605 mental health questionnaires were distributed among the study participants (319 in the network-sample and 286 in the registry based sample). Of those, 395 questionnaires were returned. Ten participants did not fulfill the inclusion criteria, and 30 respondents had to be excluded from the analyses because of too many missing values (>30%) in the Frankfurt Acculturation Scale (FRACC). In total, 328 individuals of Turkish origin were included in the study. Nearly half of them (48.5%) were recruited by the register-based approach.

A comparison of the respondents and the non-respondents within the register-based approach demonstrated that the non-respondents (N=1197) were significantly younger than the study participants (39.0 years, SD=13.1 vs. 40.9 years, SD=11.7; p=.034), and less frequently women (46.2% vs. 57.0%; p=.008).

### Socio-Demographic and Migration-Specific Data

In **Table 1**, the socio-demographic characteristics for women and men of Turkish origin and the total study sample are presented. Of 328 participants included in the present study, 201 (61.3%) were female. The average age of the participants was 41.6 years (SD=11.3). Most of them were married, had a secondary/vocational education and a monthly household income of 1,250–2,500 € and were unemployed. Women were less frequently in a partnership, were more frequently separated/divorced and unemployed and had more frequently a low education status in relation to men. Among men twice as many persons were job seeking than among women.

**Table 1:** Socio-demographic characteristics of female and male migrants of Turkish origin and the total sample.

Variable	Women(n=201)	Men(n=127)	Total(N=328)
**Age**			
M (SD)	41.2 (11.0)	42.1 (11.8)	41.6 (11.3)
Range	20 – 69	20 – 68	20 – 69
**Age group,** n (%)			
20–35	70 (34.8)	35 (27.6)	105 (32.0)
36–55	107 (53.2)	77 (60.6)	184 (56.1)
≥56	24 (11.9)	15 (11.8)	39 (11.9)
No data	–	–	–
**Partnership,** n (%)			
Yes	156 (77.6)	112 (88.2)	268 (81.7)
No	45 (22.4)	15 (11.8)	60 (18.3)
No data	–	–	–
**Marital status,** n (%)			
Single	19 (9.5)	20 (15.7)	39 (11.9)
Married	143 (71.1)	98 (77.2)	241 (73.5)
Separated/divorced	32 (15.9)	8 (6.3)	40 (12.2)
Widowed	5 (2.5)	0 (0.0)	5 (1.5)
No data	2 (1.0)	1 (0.8)	3 (0.9)
**Education,** n (%)			
No degree	8 (4.0)	3 (2.4)	11 (3.4)
Primary school	71 (35.3)	27 (21.3)	98 (29.9)
Middle school	43 (21.4)	39 (30.7)	82 (25.0)
Secondary/vocational school	51 (25.4)	55 (43.3)	106 (32.3)
No data	28 (13.9)	3 (2.4)	31 (9.5)
**Employment status,** n (%)			
Employed	51 (25.4)	61 (48.0)	112 (34.1)
Unemployed (household)	102 (50.7)	20 (15.7)	122 (37.2)
Job seeking	19 (9.5)	24 (18.9)	43 (13.1)
Pensioner	18 (9.0)	17 (13.4)	35 (10.7)
Other	8 (4.0)	5 (3.9)	13 (4.0)
No Data	3 (1.5)	–	3 (0.9)
**Monthly household income,** n (%)			
<1,250€	44 (21.9)	32 (25.2)	76 (23.2)
1,250–2,500€	87 (43.3)	60 (47.2)	147 (44.8)
>2,500€	31 (15.4)	27 (21.3)	58 (17.7)
No Data	39 (19.4)	8 (6.3)	47 (14.3)

**Table 2** reports the migration-related data of the participants. The large part of the sample (women as well as men) belongs to the first generation of migrants and has the Turkish citizenship. Most of the Turkish migrants came as young adults to Germany, are living in the destination country for 15-24 years and assess their proficiency of the German language as moderate [however, 93% of the second generation vs. 31.8% of the first generation report (very) good language proficiency or even German as mother tongue (data not shown)]. The distribution of migration-related characteristics is similar for both genders.

**Table 2:** Migration-related characteristics of female and male migrants of Turkish origin and the total sample.

Variable	Women(n=201)	Men(n=127)	Total(N=328)
**Born in Germany, n** (%)			
Yes	36 (17.9)	22 (17.3)	58 (17.7)
No	164 (81.6)	105 (82.7)	269 (82.0)
No data	1 (0.5)	–	1 (0.3)
**Length of residence in Germany, groups,** n (%)			
<5	6 (3.0)	7 (5.5)	13 (4.0)
5–14	26 (12.9)	17 (13.4)	43 (13.1)
15–24	50 (24.9)	34 (26.8)	84 (25.6)
25–34	29 (14.4)	21 (16.5)	50 (15.2)
>=35	38 (18.9)	20 (15.7)	58 (17.7)
No data	52 (25.9)	28 (22.0)	80 (24.4)
**Age at immigration, groups,** n (%)			
<6	9 (4.5)	2 (1.6)	11 (3.4)
6–12	14 (7.0)	11 (8.7)	25 (7.6)
13–17	35 (17.4)	21 (16.5)	56 (17.1)
18–25	64 (31.8)	42 (33.1)	106 (32.3)
26–39	24 (11.9)	22 (17.3)	46 (14.0)
>=40	3 (1.5)	1 (0.8)	4 (1.2)
No Data	52 (25.9)	28 (22.0)	80 (24.4)
**Citizenship,** n (%)			
German	30 (14.9)	22 (17.3)	52 (15.9)
Turkish	161 (80.1)	101 (79.5)	262 (79.9)
Dual citizenship	5 (2.5)	2 (1.6)	7 (2.1)
No Data	5 (2.5)	2 (1.6)	7 (2.1)
**Language proficiency,** n (%)			
German as mother tongue	11 (5.5)	10 (7.9)	21 (6.4)
Very good	31 (15.4)	17 (13.4)	48 (14.6)
Good	38 (18.9)	29 (22.8)	67 (20.4)
Moderate	64 (31.8)	48 (37.8)	112 (34.1)
Little	43 (21.4)	16 (12.6)	59 (18.0)
Bad	7 (3.5)	4 (3.1)	11 (3.4)
No Data	7 (3.5)	3 (2.4)	10 (3.0)

### Acculturative Styles

Based on the gender-stratified median split of the FRACC, 21.9% (n=44) of the women with Turkish origin were categorized as integrated, 28.4% (n=57) as assimilated or separated, respectively, and 21.4% (n=43) belonged to the marginalization group. In men, 26.8% were classified as integrated (n=34), 26.0% (n=33) as assimilated or separated, respectively, and 21.3% (n=27) were allocated to the marginalization group.

### Acculturative Styles and German Language Proficiency

When regarding the association between the acculturative styles and the language proficiency (data not shown), 85.2% (n=46) of the separated women, 72.1% (n=31) of the marginalization group, 61.0% (n=25) of the integrated and 21.4% (n=12) of the assimilated women reported bad to moderate language proficiency (in comparison with good/very good proficiency/German as mother tongue). The difference was significant (χ^2^(3)=51.01, p<0.001). In men, the respective proportions were: 96.9% (n=31) for separation, 53.8% (n=14) for marginalization, 45.5% (n=15) for integration and 24.2% (n=8) for assimilation. The difference was significant (χ^2^(3)=36.49, p<0.001).

### Frequency and Severity of Depressive Symptoms

The cut-off-value of ≥10 for the PHQ-9 was achieved by 33.2% (n=64) of the women and 26.4% (n=32) of the men (χ^2^(1)=1.58, p=0.209, φ=0.071). The mean age-adjusted PHQ-9 score for the female migrants was M=7.81 (SD=6.42) and for the males M=6.70 (SD=6.41). The ANCOVA with severity of depressive symptoms as dependent variable, gender as independent variable and the covariate age revealed no significant association between depressive symptoms and gender [F(1)=2.23, p=0.137, η^2^=0.007] (**Table 3**). Also no significant inter-generation difference was observed among women, however the significance level was only slightly failed [first generation: M=8.24, SD=6.42 vs. second generation: M=5.82, SD=6.82; F(1)=3.57, p=0.060, η^2^=0.018]. Men who have immigrated to Germany (M=7.50, SD=6.62) demonstrated a significantly higher age-adjusted degree of depressive symptoms than men who were born in Germany [M=3.30, SD=7.25; F(1)=5.75, p=0.018, η^2^=0.046].

**Table 3:** Differences in degrees of depressive symptoms and life satisfaction among persons of Turkish origin by gender and migration generation (gender-stratified).

	**Depressive symptoms**				**Life satisfaction**			
	M* (SD)	**F**	**p**	**η^2^**	**M (SD)**	**F**	**p**	**η^2^**
**Gender**		2.23	0.137	0.007		0.36	0.547	0.001
Female	7.81 (6.42)				3.03 (0.81)			
Male	6.70 (6.41)				3.09 (0.82)			
Female	7.56 (6.22)+	3.27	0.072	0.012	3.04 (0.82)+	0.99	0.322	0.004
Male	6.14 (6.23)+				3.14 (0.81)+			
**Migration Generation^#^**								
**Women**		3.57	0.060	0.018		8.58	0.004	0.043
First	8.24 (6.42)				2.95 (0.79)			
Second	5.82 (6.82)				3.41 (0.85)			
**Men**		5.75	0.018	0.046		8.71	0.004	0.066
First	7.50 (6.62)				2.97 (0.85)			
Second	3.30 (7.25)				3.63 (0.93)			

*Age-adjusted; ^#^first migration generation = persons with Turkish migration background immigrated to Germany, second migration generation = persons with Turkish migration background born in Germany; M, mean; SD, standard deviation; η^2^, effect size; + jobseeking persons were excluded from the analysis.

When controlling the impact of the joblessness on depressive symptoms by exclusion of the job-seeking persons from the analysis, women showed a trend for being more frequently depressed than men [χ^2^(1)=3.68, p=0.055, φ=0.117] and also demonstrated a trend for higher levels of depressive symptoms [F(1)=3.27, p=0.072, η^2^=0.012].

### Predictors of Severity of Depressive Symptoms

To examine the influence of acculturative styles as well as socio-demographic and migration-related variables on the severity of depressive symptoms, multiple linear regression analyses were performed separately for women and men. In females as well as in males, in the unadjusted regression model separation (women: B=3.988, 95% CI=1.487, 6.489, p=0.002; men: B=5.60, 95% CI=2.562, 8.638, p<0.001) and marginalization (women: B=3.148, 95% CI=0.475, 5.820, p=0.021; men: B=3.609, 95% CI=0.405, 6.813, p=0.028) were significantly associated with higher levels of depressive symptoms in relation to integration. The variance explained was 5.4% for women and 12.0% for men.

In the adjusted regression model (**Table 4**), the acculturative styles and the socio-demographic variables age, partnership, education level (dichotomized) and employment status (dichotomized) as well as the migration-related variables migration generation and German language proficiency were included into the model as predictors and the sum score of depressive symptoms as the criterion variable. In migrant women, separation as acculturation style (B=4.424, 95% CI=1.680, 7.168, p=0.002; reference: integration), having no partnership (B=2.558, 95% CI=0.256, 4.859, p=0.030) and lower education (B=-2.280, 95% CI=-4.537, -0.022, p=0.048) were associated with higher depression levels. The variance explained was 8.9%. In migrant men, separation as acculturation style (B=4.008, 95% CI=0.704, 7.312, p=0.018; reference: integration) and employment status (B=-3.317, 95% CI=-5.710, -0.924, p=0.007) were related to depression levels (explained variance: 17.2%).

**Table 4:** Multiple linear regression analyses of depressive symptoms (PHQ-9) for female and male migrants of Turkish origin.

Independent variables	Females (EV=8.9%)					Males (EV=17.2%)				
	Regression coefficient(95% CI)	Standard error	Beta	T	p	Regression coefficient(95% CI)	Standard error	Beta	T	p
Constant	3.789 (-1.910, 9.487)	2.885		1.313	0.191	7.173 (-0.434 – 14.780)	3.838		1.869	0.064
**Acculturation group**										
Integration	Ref.					Ref.				
Assimilation	1.225 (-1.629, 4.079)	1.445	0.087	0.848	0.398	0.417 (-2.761, 3.595)	1.604	0.028	0.260	0.795
Separation	4.424 (1.680, 7.168)	1.389	0.314	3.185	**0.002**	4.008 (0.704, 7.312)	1.667	0.270	2.404	**0.018**
Marginalization	2.690 (-0.239, 5.618)	1.483	0.174	1.814	0.072	2.570 (-0.763, 5.903)	1.682	0.162	1.528	0.129
**Age**	-0.015 (-0.116, 0.086)	0.051	-0.026	-0.295	0.768	-0.061 (-0.174, 0.052)	0.057	-0.110	-1.066	0.289
**Partnership**	2.558 (0.256, 4.859)	1.165	0.168	2.195	**0.030**	2.458 (-1.051, 5.968)	1.771	0.122	1.388	0.168
**Education**	-2.280 (-4.537, -0.022)	1.143	-0.164	-1.995	**0.048**	0.010 (-2.375, 2.395)	1.203	0.001	0.009	0.993
**Employment status**	-0.761 (-3.086, 1.565)	1.177	-0.052	-0.646	0.519	-3.317 (-5.710, -0.924)	1.207	-0.255	-2.748	**0.007**
**Generation of migration**	-2.922 (-5.926, 0.081)	1.521	-0.177	-1.922	0.056	-2.401 (-6.447, 1.645)	2.041	-0.140	-1.176	0.242
**German language proficiency**	1.718 (-0.663, 4,100)	1.206	0.133	1.425	0.156	-0.716 (-3.651, 2.219)	1.481	-0.055	-0.484	0.630

EV, explanation of variance; Ref., Reference; Partnership: (yes vs. no); Education: dichotomized: below secondary/vocational education vs. secondary/vocational education; Employment status: dichotomized: unemployed/jobseeking/pensioner/other vs. employed; Generation of migration: first vs. second; German language proficiency: dichotomized: bad/little/moderate vs. good/very good/German as mother tongue; significant predictors are marked in bold.

### Life Satisfaction (LS)

Regarding the age-adjusted mean scores of LS, both genders did not differ significantly [women: M=3.03, SD=0.81 vs. men: M=3.09, SD=0.82, F(1)=0.36, p=0.547, η^2^=0.001] (**Table 3**). This was also the case after controlling for unemployment status. Among migrant women, the ANCOVA with degree of LS as dependent variable, migration generation as independent variable and the covariate age showed a significant association between LS and the generation of migration [F(1)=8.58, p=0.004, η^2^=0.043]: the first generation migrants achieved a significantly lower age-adjusted level of LS in comparison with the second generation migrants (M=2.95, SD=0.79 vs. M=3.41, SD=0.85). The same findings were found for migrant men: [F(1)=8.71, p=0.004, η^2^=0.066]: the first generation migrants showed a significantly lower LS in relation to the second generation migrants (M=2.97, SD=0.85 vs. M=3.63, SD=0.93).

## Discussion

The first aim of the present study was to examine gender-specific differences regarding the frequency and severity of depressive symptoms and the degree of LS in migrants of Turkish origin. After controlling for unemployment status (i.e. excluding the job-seeking persons from the analysis), women showed a trend for being more frequently depressed (cut-off-value PHQ-9≥10) than men and also demonstrated a trend for higher levels of depressive symptoms. These findings are in line with the majority of studies demonstrating increased levels of depressive symptoms in women as compared with men in general and in female in relation to male migrants (37, 38). There are several possible explanations for this result. The gender gap may reflect the gender difference generally observed in the normal population showing a higher vulnerability for mental disorders in women (38). Furthermore, it may (partially) result from culturally influenced gender roles allowing women present more symptoms than men. Increased frequencies and severity of depressive symptoms among women may also be attributed to higher acculturative stress, higher burdens (e.g. employment, caring for children and elderly parents) and a worse socio-economic status in comparison with men. Besides, neurobiological factors (39), differences in coping skills and personality characteristics as well as adverse experiences in childhood (40) may explain the gender gap. Probably an interaction between multiple factors such as the ones mentioned above and also other factors not examined in this study could have contributed to the gender gap in depression.

The second central aim of this survey was the investigation of the association between acculturative styles and the severity of depressive symptoms when adjusted for socio-demographic and migration-related variables separately in women and men of Turkish origin. A major finding was the observation that separation was related to higher symptom severity in both genders in reference to integration. These results confirm previous research showing separation to be associated with higher levels of depressive symptoms in Turkish migrants compared to integration in non-clinical (15, 16) as well as in clinical samples (13) and also in other migrant collectives (41). This negative association between separation and mental health status was also found for other indicators of mental health, such as health-related quality of life (18).

There are several possible explanations for this pattern. It can be assumed that separation is connected with fewer skills (e.g., language proficiency – 85.2% of the separated women and 96.9% of the separated men in our study reported bad to moderate knowledge of the German language – these were the highest proportions among the four acculturative styles) and resources (e.g., familiarity with the healthcare system; social network) that are required to challenge successfully the demands of living in the country of destination such as acquisition of satisfactory education or positions in the employment domain or access to healthcare institutions or social services. Thus, a low adaptation to the host society may increase acculturative stress and frustration and finally lead to elevated levels of depressive symptoms (42). Some separated persons may experience – in a chronically stressful way – a kind of social defeat (43) due to inadequate competences in managing effectively and satisfactory life in the new society. A prolonged experience of outsider status may contribute to the manifestation of depressive symptoms. Social defeat has been demonstrated to be a stressor inducing depression (44, 45).

The negative relationship between separation and mental health may also be attributed to high levels of perceived discrimination in separated persons that moderate depressive symptoms. Highest degree of perceived discrimination has been found in separated (second generation) and marginalized (both generations) women of Turkish origin (46). Perceived discrimination has been shown to be related with depressive symptoms in Turkish migrants (5) as well as other migrants groups (45, 47–50). The European Social Surveys from 2006/2007 (51) and 2014 (37) confirm the association between perceived discrimination and depressive symptoms in migrants and also non-migrants.

Also other psychological or psychosocial factors may moderate the association between separation and depressive symptoms in migrants, for example self-esteem has been demonstrated to moderate the effect of perceived discrimination on depression (50). In some studies, an association between a higher level of acculturation and better performance on cognitive function tests has been detected (52). In separated and marginalized migrants, a higher proportion of persons with a low education level has been observed than in integrated or assimilated individuals (16).

Finally, due to the cross-sectional design of the present study, a reverse relationship between separation and depression cannot be excluded. It could also be possible that depressive persons prefer separation as acculturative style. Prospective studies are needed to examine the causal direction of this association.

Apart from separation that was found to be significantly associated with depressive symptoms for both sexes, also gender-specific significant predictors for the severity of depressive symptoms were identified in the present study, namely, having no partnership among women and not being employed in men. These different results for both genders may reflect gender-specific cultural expectations towards gender roles which play a crucial role especially in socio-centric societies such as the Turkish one. The traditional Turkish culture is characterized by a strict gender and generation hierarchy and a strong family cohesion/integration and close interpersonal relationships (53). A woman is expected to be primarily responsible for the household, child-rearing, and relationship maintenance, and a man should provide financial support for his family. Thus, having no partnership in women and unemployment in men contradicts the perception of feminity and masculinity, respectively, in the Turkish culture and may decrease the self-esteem as a woman or a man, evoke feelings of shame or guilty and finally depressive symptoms.

In our investigation, the impact of assimilation on the manifestation of depressive symptoms did not differ significantly from integration. This result is consistent with some previous findings (13), while others have shown integration to be the most favorable acculturation style for mental health (14). A possible reason for the similar effect of integration and assimilation may be due to the migration policy in Germany. In the context of migration it is important to emphasize that the degree of acculturation to the host society depends not only on the migrants themselves but also on a country´s national migration policy that constitutes institutional support and acceptance of migrants or contributes to their social exclusion and barriers (54). In Germany, in many decades of the 20^th^ century a model aiming at a sociocultural assimilation of migrants was followed by German authorities. Furthermore, orientation on the host culture is more important for an adequate adaptation than maintaining the cultural identity of the heritage culture. In some contexts, assimilation may even be more beneficial, e.g., for academic achievements, while integration is favorable for indicators of psychological adaptation (55). In that context, the influence of perceived discrimination which seems to be higher in second generation migrants is worthy to be mentioned, especially if they try to integrate their cultural heritage after following an assimilation strategy or perceive “transparent barriers” in their career or at the level of political participation. El-Mafaalani has presented these findings as the so-called “integration paradox” (56) and could explain new acculturative stressors if more participation is desired and could even lead to new contextual separation strategies during the life course.

Contrary to our expectations, marginalization was not significantly related to higher severity of depressive symptoms as compared with integration. However, in women marginalization revealed a tendency for higher depression levels.

The low explained variance in the regression analyses for depression is worth to be mentioned: 8.9% (women) and 17.2% (men). It may be postulated that other essential protective and risk factors not examined in the present study, such as sense of coherence (6), self-esteem (50), social support (45) or (perceived) discrimination (5) may also substantially influence the manifestation of depressive symptoms.

In terms of inter-generational differences for LS, in the present study lower age-adjusted levels of LS were observed in the first migrant generation compared to the second. The research on LS in dependence of migration generation is inconclusive. Some surveys confirm the result found in our investigation [e.g., Fugl-Meyer et al. (22)], while others report contrary results [e.g., Knies et al. (21)]. The first generation may be faced with many substantial losses (e.g., of social ties and support, social status and professional degradation) and in some cases with higher acculturative stress (e.g., learning a new language) than it is the case for the second generation that is already socialized in the new country. In addition, in the second generation more skills (in particular language proficiency, familiarity with the health care system) and less barriers may be mostly postulated that contribute to a successful sociocultural adaptation and – as a result – a higher LS. Furthermore, in the second generation the identification with the host country is probably higher than in the migrants that have left their home country. Identification with the host country have been revealed to be predictors of higher LS (57).

### Strengths and Limitations

A strength of the present survey is the relatively large sample size that allowed stratification for gender to conduct separate analyses for women and men. Another advantage of this study was the inclusion of respondents with insufficient knowledge of the German language due to the community-orientated recruitment strategy and the bilingual questionnaire.

The results should be viewed in the light of some limitations. The sample is not strictly population based because of the network sampling approach in parts of the sample and the low response rate among the register-based sample (28). Thus, the findings cannot be generalized to the general population of Turkish migrants in Germany or to other migrant collectives. Another limitation is the cross-sectional study design that does not allow for drawing causal conclusions regarding the influence of the variables measured. Besides, the classification of the participants into the four acculturation groups was based on the medians for the acculturation strategy orientation on the culture of origin and the host culture observed in the present sample due to the lack of (validated) cut-off or norm values. Thus, no extern criteria exist and the categorization may vary depending on the medians in different samples. Furthermore, one can call into question if the four acculturation styles proposed by Berry cover the full spectrum of all existing strategies given that people may adapt different strategies depending their personal experiences and changing societal constraints and expectations. Also the low Cronbach’s Alpha for the Turkish version of the subscale orientation on the host culture (α = 0.53) may decrease the reliability of the measure of acculturation. Finally, the significant differences between responders and non-responders (non-responders were younger and less frequent women) could also present a selection bias so that it can be assumed that persons participated in the study may belong to more vulnerable individuals (probably mostly unemployed persons with a low socio-economic status) with a higher tendency for the manifestation of depressive symptoms and a lower LS than the non-responders.

## Conclusion

The results of this study indicate that the acculturative style separation is associated with higher levels of depressive symptoms (for both genders) in migrants of Turkish origin living in Germany as compared with integration. Therefore, gender-sensitive health promotion programs should target separated migrants to improve their integration into the German society. In addition, our findings suggest gender-specific protective and risk factors for depression. Culture-specific factors should be appropriately taken into consideration in prevention and intervention programs. Special attention should be given to identify depressive symptoms in female migrants to improve the LS (e.g., screening instruments and special trainings for general practitioners).

Further research should investigate the role and interrelationships of further migration- and acculturation-related stressors (e.g., perceived discrimination) and non-migration-related factors (e.g., socio-economic status) as well as culture-specific aspects (e.g., illness-specific beliefs) in the context of depressive symptoms and LS in Turkish migrants and other migrant collectives. It remains an important issue for public health research and policies to investigate mental health of migrants in representative surveys with special attention to gender-specific differences.

## Data Availability Statement

The datasets generated for this study are available on request to the corresponding author.

## Ethics Statement

The studies involving human participants were reviewed and approved by Ethics Committee of the Medical Faculty of the University of Duisburg-Essen. The patients/participants provided their written informed consent to participate in this study.

## Author Contributions

EM and YE designed and conducted the study, EM analyzed the data and wrote this manuscript. YE provided feedback and mentorship on each stage of the research design and implementation, including a full review and provision of feedback on the final manuscript. TB, ND, SM, and K-HJ contributed to manuscript writing.

## Conflict of Interest

The authors declare that the research was conducted in the absence of any commercial or financial relationships that could be construed as a potential conflict of interest.

## Abbreviations

ANCOVA, Analysis of covariance; B, linear regression coefficient; CI, Confidence interval; CO: Culture of origin; DSM-IV, The fourth edition of the Diagnostic and Statistical Manual of Mental Disorders; η^2^, Eta square (effect size); FRACC, Frankfurt Acculturation Scale; HC, Host culture; LS, Life satisfaction; M, Mean; OECD, Organization for Economic Cooperation and Development; PHQ-9, Patient Health Questionnaire: depressive symptom severity scale; SD, Standard deviation.
